# Reconfiguration of the Brain Functional Network Associated with Visual Task Demands

**DOI:** 10.1371/journal.pone.0132518

**Published:** 2015-07-06

**Authors:** Xue Wen, Delong Zhang, Bishan Liang, Ruibin Zhang, Zengjian Wang, Junjing Wang, Ming Liu, Ruiwang Huang

**Affiliations:** 1 Center for the Study of Applied Psychology, Key Laboratory of Mental Health and Cognitive Science of Guangdong Province, School of Psychology, South China Normal University, Guangzhou, China; 2 Department of Radiology, Guangdong Provincial Hospital of Chinese Medicine, Guangzhou, China; 3 Guangzhou University of Chinese Medicine Postdoctoral Mobile Research Station, Guangzhou, China; National Yang-Ming University, TAIWAN

## Abstract

Neuroimaging studies have demonstrated that the topological properties of resting-state brain functional networks are modulated through task performances. However, the reconfiguration of functional networks associated with distinct degrees of task demands is not well understood. In the present study, we acquired fMRI data from 18 healthy adult volunteers during resting-state (RS) and two visual tasks (i.e., visual stimulus watching, VSW; and visual stimulus decision, VSD). Subsequently, we constructed the functional brain networks associated with these three conditions and analyzed the changes in the topological properties (e.g., network efficiency, wiring-cost, modularity, and robustness) among them. Although the small-world attributes were preserved qualitatively across the functional networks of the three conditions, changes in the topological properties were also observed. Compared with the resting-state, the functional networks associated with the visual tasks exhibited significantly increased network efficiency and wiring-cost, but decreased modularity and network robustness. The changes in the task-related topological properties were modulated according to the task complexity (i.e., from RS to VSW and VSD). Moreover, at the regional level, we observed that the increased nodal efficiencies in the visual and working memory regions were positively associated with the increase in task complexity. Together, these results suggest that the increased efficiency of the functional brain network and higher wiring-cost were observed to afford the demands of visual tasks. These observations provide further insights into the mechanisms underlying the reconfiguration of the brain network during task performance.

## Introduction

The human brain is a highly complex network [1–3], in which anatomically distributed brain regions functionally interact for information communication [4]. The complex intrinsic brain network underpins brain function and dynamically reconfigures to sustain various mental states [1]. Although the diversity of the intrinsic brain network topological organization has been widely explored [5, 6], the dynamic reconfiguration of the brain functional network associated with task demand remains elusive.

The graph theory can be used to characterize the organization of intrinsic brain network [7]. Previous studies have demonstrated the small-world properties in the human brain functional network [8, 9], i.e., the highly efficient topological organization of the brain network is characterized by high clustering and low path length [10]. In addition, the brain functional networks are also modularly organized [11, 12] and contain a subset of highly connected hubs [13]. These topological attributes of the intrinsic brain network have been shown to be crucial for efficient brain communication. In particular, the brain functional network during task performance is strongly shaped by the intrinsic network architecture during rest [14]. Meanwhile, many studies have also shown that the brain functional network can be modulated through various factors, such as brain disorders [15, 16], drug addiction [10, 17], age [10, 18], sex [19], and intelligence [20]. The fact that the intrinsic network is modulated by these factors reflects the nature of the dynamically adaptive reconfiguration of brain networks. Indeed, the dynamically adaptive reconfiguration of brain functional networks during active task performance has been demonstrated in several studies. For example, the reconfiguration of the brain functional network has been investigated using different task performances, such as finger movements [21], music perception [22], visual stimulation [23], auditory stimulation [24], and working memory [25, 26]. Compared with the intrinsic brain network, these studies collectively suggested that the topological organization of brain network could be dynamically modified by the task performances. Despite this, it is still largely unclear how the complex topology of human brain network changes with increased cognitive demands.

To fill this gap, the present study aimed to explore the dynamic reconfiguration of the topological organization of the brain functional network associated with different levels of cognitive demands in visual tasks. Toward this end, we acquired fMRI data from 18 healthy subjects under three conditions: resting-state (RS), visual stimulus watching (VSW), and visual stimulus decision (VSD). The brain functional network for each condition was constructed, and the network properties were measured using graph theory analysis [7]. In addition, we compared the network properties among these three conditions and investigated the reconfiguration of the brain functional network associated with visual tasks. Moreover, we validated the results of the present study using additional analyses [27, 28].

## Materials and Methods

### Participants

Eighteen healthy volunteers (age = 23.6 ± 1.65 years, 10 males) participated in the present study. All participants were enrolled in South China Normal University, Guangzhou, China. The exclusion criteria included psychiatric or neurological illness, head trauma, and any implant, device, or object in the body. All the participants provided written informed consent, and the protocol was approved through the Research Ethics Review Board of South China Normal University.

### Task manipulation

Three cognitive conditions (i.e., RS, VSW, and VSD) were used in the present study, and the participants with normal or corrected-to-normal vision, were fitted with earplugs and provided a hand-fitted keyboard for each condition. The functional images for these three conditions were acquired in a single session. Briefly, each participant was instructed to lie still with eyes closed and not fall asleep during the RS scan, and we acquired 240 volume images in 8 min. Under the VSW condition, each participant was instructed to watch the block stimuli, presented as a series of pictures displayed on the center of a screen in random order. In each block, the visual stimulus lasted 26.4 s, with alternating 12-s intervals of cross cursor epoch. The scan time was 10.2 min for the VSW. For VSD, a one-back task was applied, in which the participant was asked to press the button on the hand-fitted keyboard to indicate whether the presented picture was the same as the one before it. The scan time was 16.8 min. The flow charts of VSW and VSD are presented in Fig A in S1 Appendix (Supplementary Materials). For each participant, the scans were counterbalanced across participants to minimize the effect of condition order.

### Data acquisition

All MRI data were obtained on a 3 T Siemens Trio Tim MR scanner with a 12-channel phased array head coil in South China Normal University. The fMRI data were acquired using a gradient-echo echo-planar imaging (EPI) sequence with the following parameters: TR = 2000 ms, TE = 30 ms, Flip angle = 90°, matrix = 64 × 64, FOV = 220 × 220 mm^2^, thickness/gap = 3.5/0.8 mm, and 32 axial slices covering the whole brain. In addition, 3D high-resolution brain structural images were obtained using a 3D T1-weighted MP-RAGE sequence with the following parameters: TR = 1900 ms, TE = 2.52 ms, Flip angle = 9°, matrix = 256 × 256, FOV = 230 × 230 mm^2^, thickness = 1.0 mm, and 176 sagittal slices.

### Data preprocessing

All MRI data were processed using DPARSF_V2.0 [29] based on the SPM8 toolkit (http://www.fil.ion.ucl.ac.uk/spm/). Consistent data processing procedures were applied for the three cognitive conditions. The fMRI data of the three conditions for each subject were processed separately. To minimize the effects of the length of the time series on network analysis [30], we used the first 8 min fMRI data of VSW and VSD to yield 240 time points that are comparable to the RS condition. As described previously [17], the following procedures were adopted. First, the first 10 volume images were removed from the fMRI data for scanner stabilization and participant adaptation to the environment. Subsequently, slice timing and realignment were performed to correct for the acquisition time delay and head motions, respectively. We assessed the head motion of each participant, and no participant was excluded according to these criteria (i.e., the head motion in any direction was not more than 1.5 mm or 1.5°). We adopted the maximum head motion of the time series to represent the head motion profile in any direction for each condition of each subject, and used repeated measures ANOVA to analyze the differences of head motion in any direction among the three conditions. Notably, the head motion profiles were matched among the three conditions (i.e., RS, VSW and VSD) (*p* > 0.118 in any direction). The acquired functional images were further spatially normalized to a standard MNI template and resampled to a voxel size of 3×3×3 mm^3^. Here, we did not perform spatial smoothing, consistent with several previous studies [10, 31]. To reduce low frequency drift and the physiological noise of fMRI data, the linear detrend was removed, and 0.01Hz high-pass filtering was used. Moreover, we regressed out the nuisance covariates, including the 6 head motion parameters and the white matter and cerebrospinal fluid (CSF) signals. The global signal was not regressed out due to recent debates [32–34].

### Construction of brain functional networks

To define the node of the functional network, we applied a functional template as proposed in a previous study [35]. This functional template (referred to as “Fun160” in the present study) comprised 160 regions of interest (ROIs) (i.e., putative functional nodes), spanning across the cerebral cortex, subcortical structures, and the cerebellum. The set of ROIs were generated around the peak coordinates previously identified from meta-analysis data of multiple brain functions using 10mm diameter spheres [35]. Although the functional template couldn’t completely deal with individual difference, it was appropriated for defining the nodes of functional networks given that it has been broadly applied in previous brain network studies [6, 35–37]. The ROIs of the template are listed in Table A in S1 Appendix. As previously described [17], the time series for each ROI was extracted by averaging the signals of all voxels within that region. For each cognitive condition of each subject, a 160 × 160 correlation matrix was obtained after calculating Pearson’s correlation coefficient for the time courses between any pair of ROIs. In the present study, we adopted a binary network for further analysis, as this method is simple and easy to interpret. To obtain the binary matrix, we applied a statistical-specific threshold to the correlation matrix. Briefly, when calculating the Pearson’s correlation coefficient, the corresponding p-value of each element in the matrix was also calculated, the p-value was used to compare with a statistical threshold. Then, the matrix elements, whose corresponding p-values passed through the statistical threshold, were retained for each correlation matrix, whereas the elements were set to zero when the *p*-values did not pass through the threshold [38, 39]. We used a rigorous Bonferroni method (*p* < 0.05) to correct for multiple comparisons, that the *p*-threshold equal 0.05 divided by the total number of non-repeated elements (i.e. total number = 160×159/2). Finally, a binary functional network was obtained for each subject in each cognitive condition.

### Network analysis

#### Global parameters

We characterized the whole-brain topological organization of the brain functional networks using the following eight global parameters [7]: the average degree (*K*), clustering coefficient (*C*_p_), local efficiency (*E*_loc_), characteristic path length (*L*_p_), global efficiency (*E*_glob_), small-worldness (σ), average physical distance of functional connections (*D*_p_), and modularity (*Q*). The expression and interpretation are presented in S1 Appendix (Supplementary Materials). The *K* and *D*_p_ can be used to describe the connection density and wiring-cost of forming a network, respectively [25, 40].

#### Nodal parameters

Although the global parameters well describe the topological organization of the brain functional network, nodal analysis provides more useful information for the individual brain regions. In the present study, we used nodal efficiency (*E*_nod_) to describe the nodal topology of the brain network. The expression is listed in S1 Appendix (Supplementary Materials).

#### Network robustness analysis

In addition, we evaluated the stability of functional networks using the network robustness (*R*), characterized by the degree of tolerance against targeted attack [41, 42]. Briefly, we removed the nodes in decreasing order of the *E*_nod_ value and recalculated the size of the largest connected component after removing one node each time. The robustness is represented as the area under the curve plotted according to the component size and node number (He, Chen et al. 2008). More robust networks retain a larger connected component, even when several nodes have been removed.

### Statistical analysis

We used one-way repeated measures ANOVA models across the three cognitive conditions (RS, VSW and VSD) to detect the main effects of task demands on each of the global parameters, *K*, *C*_*p*_, *E*_*loc*_, *L*_*p*_, *E*_*glob*_, *σ*, *D*_*p*_, and *Q*. When a significant main effect was observed for a given parameter, we further performed post-hoc paired *t*-tests (Bonferroni correction for post-hoc comparisons) to determine the simple effect of the task demands. Significance was measured at *p* < 0.05.

For the nodal parameter, we performed the same ANOVA and post-hoc comparison methods to detect the main and simple effects of task demands on the *E*_nod_ in each region. Because of the large number of regions, the false discovery rate (FDR) was adopted to correct the multiple comparisons of ANOVA models across regions, and the Bonferroni correction was applied to post-hoc comparisons in regions exhibiting significant main effects. Moreover, we also compared the network robustness across the three cognitive conditions using the same procedures.

### Validation analysis

The reproducibility of our results was validated using the following procedures. We employed two additional brain templates, i.e., the AAL90 and Fun268 (Tables B and C in S1 Appendix), to define the network nodes for estimating the influence of the different parcellation schemes on our findings. In addition to the binary network analysis, we also implemented the weighted network and repeated the network analysis. The details are presented in S1 Appendix.

## Result

### Global topology

**Fig 1:**
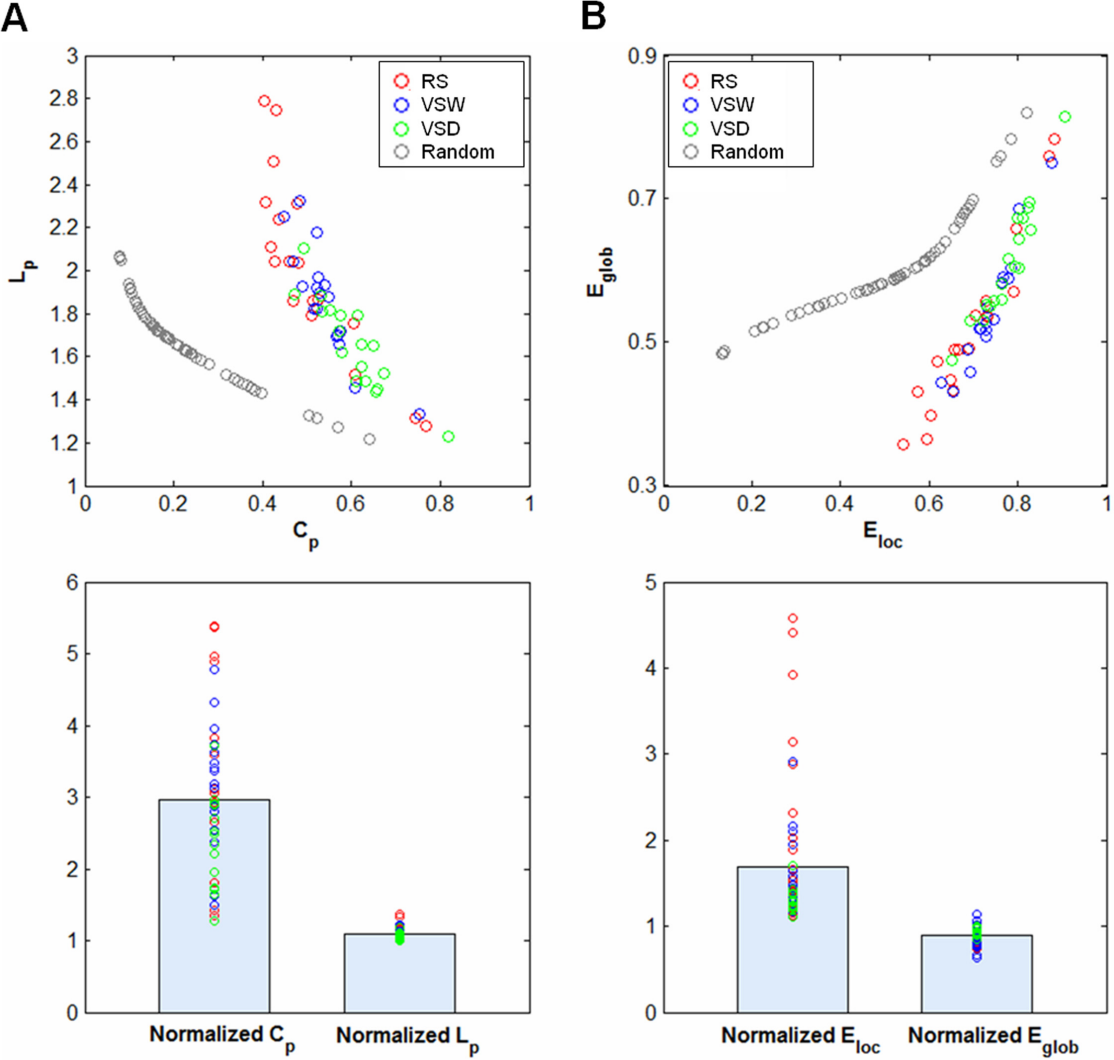
The small-world parameters and efficiency of brain functional networks. (A) The functional networks of all cognitive conditions showed a higher clustering coefficient (*C*_p_) and approximately equal characteristic path length (*L*_p_) compared with the matched random networks (top panel), resulting in normalized *C*_p_ > 1 and normalized *L*_p_ ≈ 1 (bottom panel). (B) Functional networks exhibited higher local efficiency (*E*_loc_) but approximately identical global efficiency (*E*_glob_) of parallel information transmission compared with matched random networks (top panel), resulting in normalized *E*_loc_ > 1 and normalized *E*_glob_ ≈ 1 (bottom panel). RS: resting-state, VSW: visual stimulus watching task, VSD: visual stimulus decision task.

The brain functional network for each participant across all the three cognitive conditions (RS, VSW and VSD) showed higher *C*_p_ but almost identical *L*_p_ compared with the comparable random networks (Fig 1A). The random networks were generated using Maslov’s wiring program [43], which have the same number of nodes, edges and degree distribution as the real brain functional networks. Moreover, we also observed that the brain network for each subject showed an economic small-world topology of approximately equivalent parallel information processing of *E*_glob_ but a higher fault tolerance of *E*_loc_ compared with matched random networks (Fig 1B). In addition, the small-worldness indices, *σ*, for each participant under the three cognitive conditions were larger than 1 (Table 1). These results indicated that brain functional networks preserved small-world organization under different cognitive conditions, consistent with previous studies [10, 44].

**Table 1:** Mean global parameters of brain functional networks for the three cognitive conditions (RS: resting-state; VSW: visual stimulus watching task; VSD: visual stimulus decision task).

Metric	RS	VSW	VSD	F(2,34)	*p*-value
*C*_p_	0.51 ± 0.11	0.54 ± 0.07	0.60 ± 0.08	16.49	9.8e-06
*L*_p_	2.02 ± 0.43	1.86 ± 0.25	1.66 ± 0.21	17.44	6.1e-06
*E*_loc_	0.69 ± 0.10	0.74 ± 0.06	0.78 ± 0.06	18.09	4.4e-06
*E*_glob_	0.52 ± 0.12	0.55 ± 0.08	0.61 ± 0.08	17.32	6.5e-06
*σ*	2.98 ± 0.86	2.79 ± 0.64	2.22 ± 0.50	17.53	5.9e-06
*K*	29.8 ± 22.9	31.6 ± 15.8	45.1 ± 19.2	13.40	5.1e-05
*D*_p_	69.3 ± 2.18	68.8 ± 2.34	72.0 ± 1.63	16.43	1.0e-05
*Q*	0.34 ± 0.10	0.34 ± 0.09	0.26 ± 0.06	10.95	2.1e-04
*R*	73.89 ± 6.24	76.86 ± 2.98	77.71 ± 1.93	8.17	1.3 e-03

Note: Global parameters across all three conditions were calculated for each subject and averaged as group means. *C*_p_, clustering coefficient; *L*_p_, characteristic path length; *E*_loc_, local efficiency; *E*_glob_, global efficiency; *σ*, small-worldness; *K*, average degree; *D*_p_, physical distance; *Q*, modularity; *R*, robustness. The range of σ indicated that the functional network for each subject exhibits small-world attributes in each of the three cognitive conditions (σ > 1).

**Fig 2:**
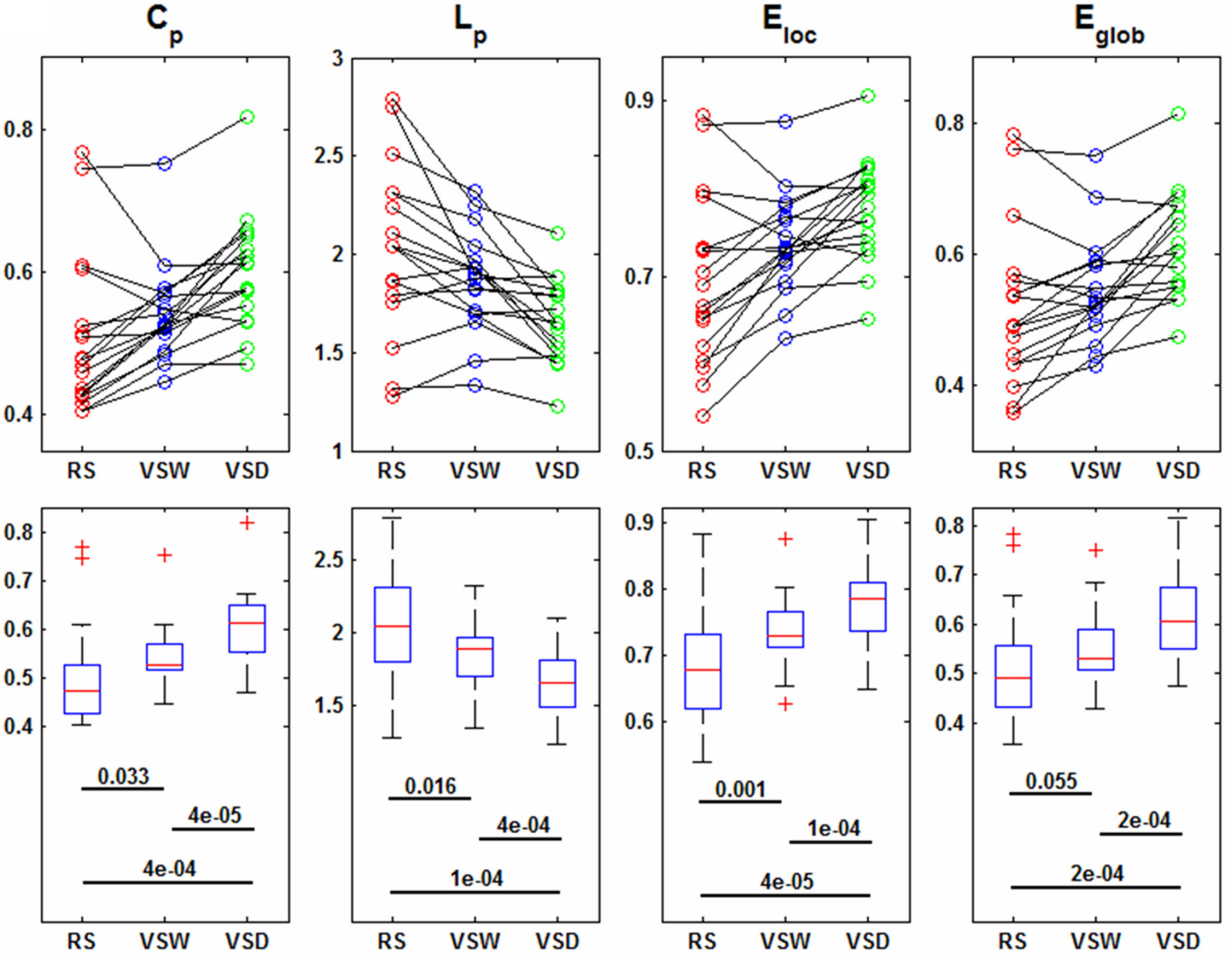
Global parameters of brain functional networks across all subjects in the three cognitive conditions. (Top row) Within-subject effects of task complexity on the clustering coefficient *C*_p_, characteristic path length *L*_p_, local efficiency *E*_loc_, global efficiency *E*_glob_. (Bottom row) Box-plots show the median, interquartile range, and range for each parameter in each condition. Each horizontal line and the associated number indicate the *p*-value of a post-hoc paired *t*-test (two-tailed). RS: resting-state, VSW: visual stimulus watching task, VSD: visual stimulus decision task.

**Fig 3:**
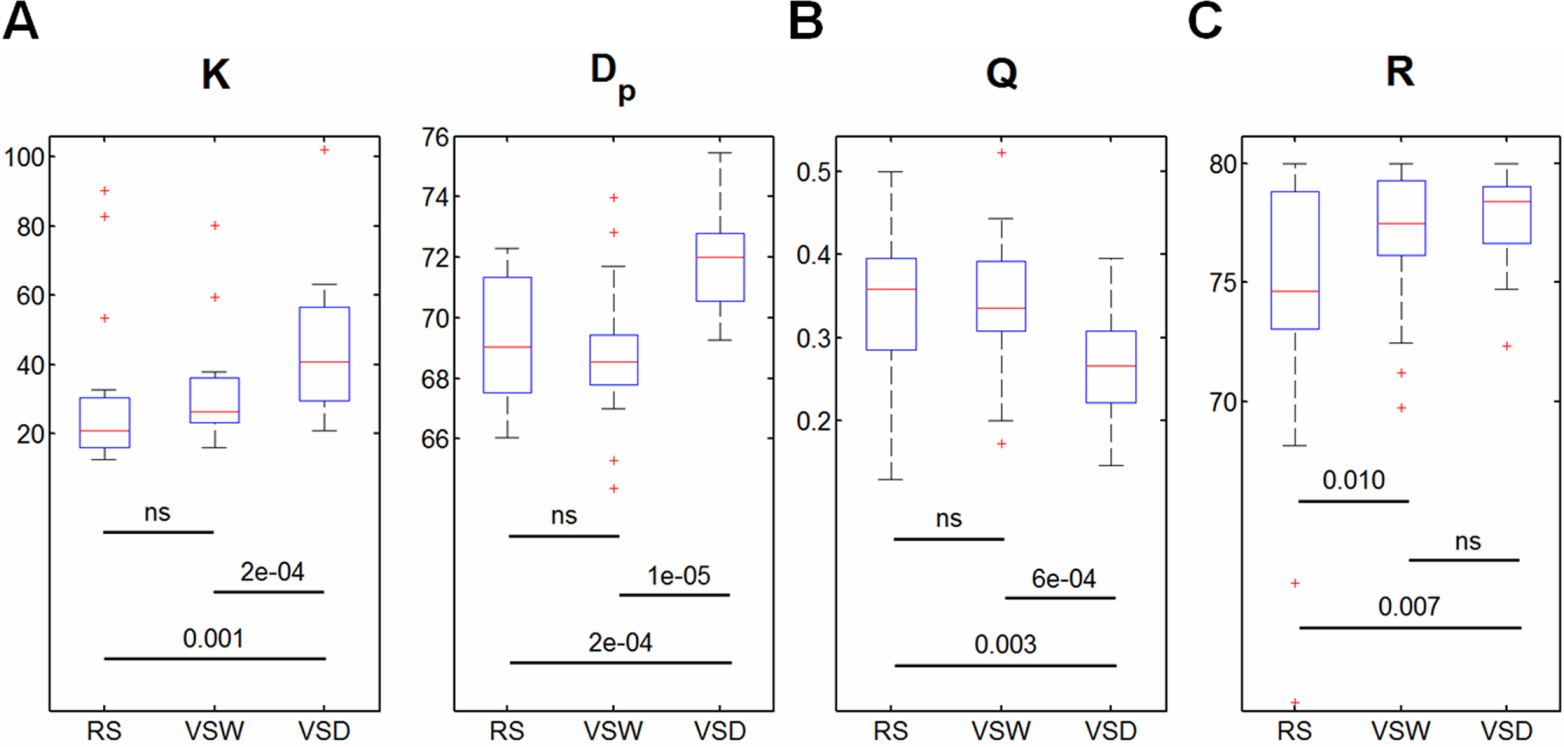
Costs, modularity, and robustness of brain functional networks across all subjects in the three cognitive conditions. Box-plots show the median, interquartile range, and range for the wiring-cost. (A) Average degree *K* and physical distance *D*_p_; (B) the modularity *Q*; and (C) the robustness *R*. Each horizontal line and the associated number indicate the *p*-value of a post-hoc paired *t*-test (two-tailed). ‘ns’ presents the *p*-value > 0.05. RS: resting-state, VSW: visual stimulus watching task, VSD: visual stimulus decision task.

Despite the small-world architecture in these brain functional networks, significant differences in the network topological properties were observed. ANOVA revealed significant main effects of task demands on all eight network global parameters across the three cognitive conditions (Table 1). In addition, post-hoc comparisons showed that *C*_p_, *E*_loc_ and *E*_glob_ monotonously increased and *L*_p_ decreased in response to the task demands ranging from RS to VSW, and then to VSD (Fig 2). This result suggested that the greater the visual task complexity, the more efficient the network, but with less small-worldness. Fig 2 also shows the individual within-subject effect of task demands on these parameters. In addition, the wiring-costs described by *K* and *D*_p_, both monotonously increased from RS to VSW, and then to VSD (Fig 3A), suggesting that the brain functional network simultaneously sacrifices a certain cost to meet the demands of complex tasks. Moreover, we observed that the greater the visual task complexity, the less modularity (i.e., decrease of *Q*) in the functional networks (Fig 3B).

### Nodal topology

**Fig 4:**
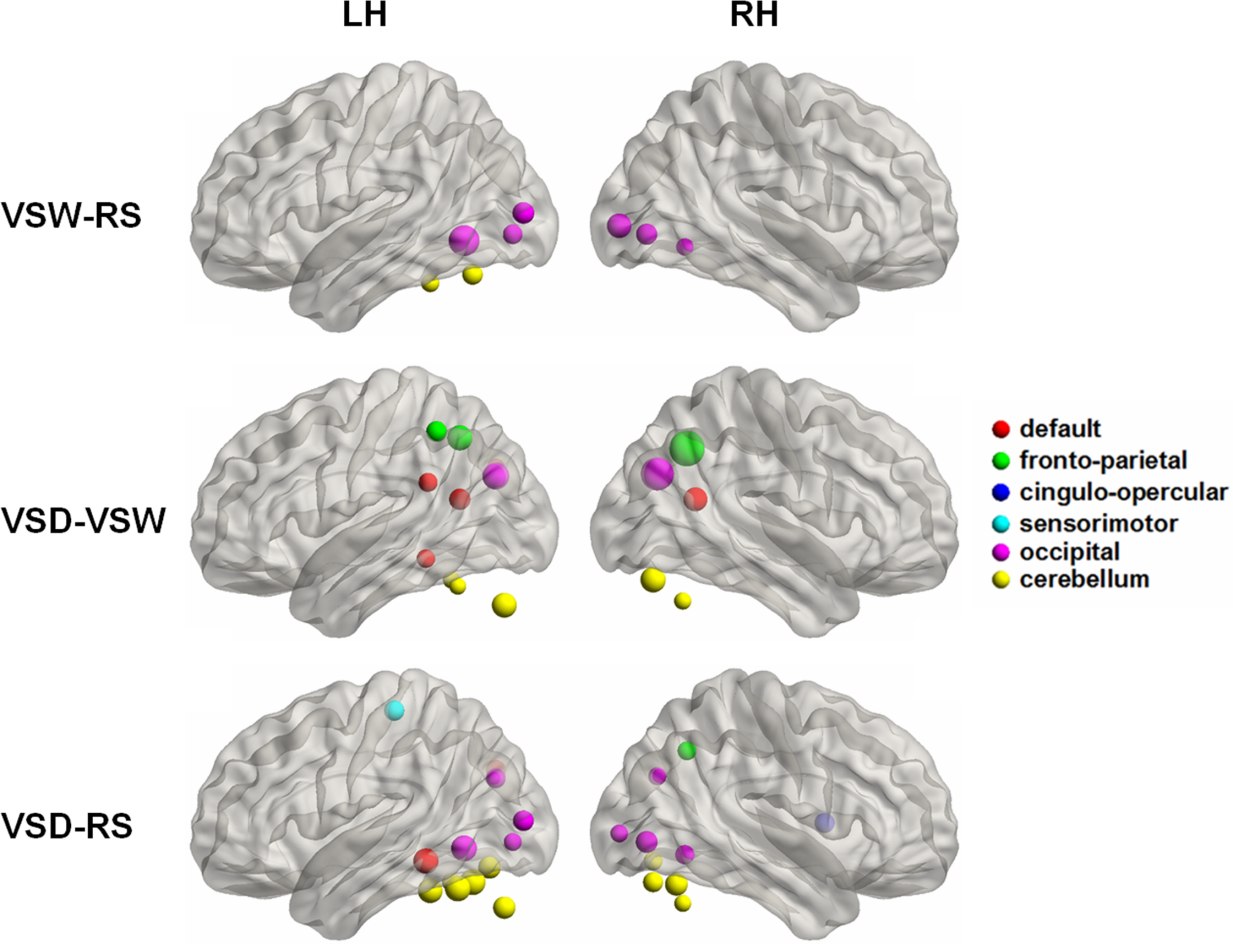
Surface visualization of the brain regions exhibiting significant between-state differences in nodal efficiency for the three comparisons, VSW-RS, VSD-VSW, and VSD-RS. The node size is proportional to the relative significance of each between-state comparison (two-tailed paired *t*-test, *p* < 0.05, FDR correction). The node colors indicate the node belonging to the six different modules according to the brain template [35]. Uniformly, we detected significantly increased nodal efficiency between each of the three comparisons, VSW-RS, VSD-VSW, and VSD-RS. RS: resting-state, VSW: visual stimulus watching task, VSD: visual stimulus decision task. LH (RH), left (right) hemisphere.

At the nodal level, the significant effects of task demands on nodal efficiencies, *E*_nod_, were observed in some brain regions. A total of 29 regions were survived using the ANOVA to indicate differences across the three cognitive conditions (*p* < 0.05, FDR corrected). Post-hoc comparisons revealed that 9 brain regions showed significantly increased *E*_nod_ in VSW compared with RS, and most of these regions (6 out of 9) were located in the occipital cortex (Fig 4). In addition, 24 regions showed significantly increased *E*_nod_ in VSD compared with RS, including 8 occipital regions and 10 cerebellum areas (Fig 4). Moreover, the consistently enhanced *E*_nod_ in VSD was primarily observed in 5 default network regions, 5 cerebellum areas, and 3 frontal-parietal regions compared with VSW (Fig 4). Notably, no region showed significantly decreased *E*_nod_ in the three pair comparisons (i.e., VSW-RS, VSD-RS, and VSD-VSW), likely reflecting the enhanced local and global efficiencies from RS to VSW, and then to VSD, as described above.

### Network robustness

The main effect of task demands on network robustness is significant (Table 1). We observed that the robustness increased with increasing task demands (progression from RS to VSW and then to VSD) (Fig 3C). This result suggested that the brain functional networks were more resilient to targeted attack (removal of nodes) under conditions of higher task demands.

### Reproducibility

Table 2 shows the reproducibility of the global parameters and robustness in the validation analysis. To quantitatively evaluate the reproducibility between the main analysis and each validation analysis, we estimated the reproducibility index as the ratio between the number of consistent comparisons and the total comparisons (the number is 36, including 9 ANOVA results and 27 post-hoc comparison results). The values for the reproducibility index were 33%, 81%, and 83% for AAL90, Fun268, and the weighted network, respectively.

**Table 2:** Validation analyses of the effects of task complexity on the global parameters derived from the binary networks in different brain templates (the AAL90 and Fun268 templates) and the weighted network in the Fun160 template.

	*C*_p_	*L*_p_	*E*_loc_	*E*_glob_	*σ*	*K*	*D*_p_	*Q*	*R*
**AAL90**									
F(2,34)	**3.68** ^a^	**3.55** ^a^	**3.40** ^a^	**3.97** ^a^	3.04 ^ns^	**3.96** ^a^	2.48 ^ns^	2.08 ^ns^	0.60 ^ns^
0052	↓ ^ns^	↑ ^ns^	↓ ^ns^	**↓** ^ns^	—	**↓** ^ns^	—	—	—
VSW vs. VSD	**↑** ^a^	**↓** ^a^	**↑** ^a^	**↑** ^a^	—	**↑** ^a^	—	—	—
RS vs. VSD	↑ ^ns^	↓ ^ns^	↑ ^ns^	↑ ^ns^	—	↑ ^ns^	—	—	—
**Fun268**									
F(2,34)	**14.8** ^b^	**13.3** ^b^	**12.6** ^b^	**13.4** ^b^	**19.9** ^b^	**13.0** ^b^	**21.8** ^b^	**11.0** ^b^	2.16 ^ns^
RS vs. VSW	↑ ^ns^	↓ ^ns^	↑ ^ns^	**↑** ^ns^	**↓** ^ns^	**↑** ^ns^	**↓** ^ns^	**↑** ^ns^	—
VSW vs. VSD	**↑** ^b^	**↓** ^b^	**↑** ^b^	**↑** ^b^	**↓** ^b^	**↑** ^b^	**↑** ^b^	**↓** ^b^	—
RS vs. VSD	**↑** ^b^	**↓** ^b^	**↑** ^b^	**↑** ^b^	**↓** ^b^	**↑** ^b^	**↑** ^b^	**↓** ^b^	—
**Weighted**									
F(2,34)	**16.1** ^b^	**19.9** ^b^	**23.3** ^b^	**18.2** ^b^	**13.7** ^b^	**13.1** ^b^	1.27 ^ns^	**11.5** ^b^	**6.74** ^b^
RS vs. VSW	↑ ^ns^	**↓** ^b^	**↑** ^b^	↑ ^a^	**↓** ^ns^	**↑** ^ns^	—	**↓** ^ns^	**↑** ^a^
VSW vs. VSD	**↑** ^b^	**↓** ^b^	**↑** ^b^	**↑** ^b^	**↓** ^b^	**↑** ^b^	—	**↓** ^b^	**↑** ^ns^
RS vs. VSD	**↑** ^b^	**↓** ^b^	**↑** ^b^	**↑** ^b^	**↓** ^b^	**↑** ^b^	—	**↓** ^b^	**↑** ^a^

Note: The validation of the nodal analysis was not performed due to the incompatibility across different brain templates. The bold text represents the consistent results compared with the main findings.—represents non-applicability because the corresponding result of ANOVA is not significant (*p* > 0.05). ↑ represents RS < VSW, VSW < VSD, and RS < VSD, respectively; ↓ represents the contrary. *C*_p_, clustering coefficient; *L*_p_, characteristic path length; *E*_loc_, local efficiency; *E*_glob_, global efficiency; *σ*, small-worldness; *K*, average degree; *D*_p_, physical distance; *Q*, modularity; *R*, robustness. RS: resting-state, VSW: visual stimulus watching task, VSD: visual stimulus decision task.

^a^ 0.01 ≤ *p*<0.05

^b^ *p*<0.01

^ns^, Nonsignificant (*p*>0.05)

## Discussion

In the present study, we investigated the topological organization of brain functional networks associated with two visual task conditions and a resting state using graph theoretical analysis. The main findings can be summarized as: 1) the small-world attributes were qualitatively preserved in the brain functional networks across the three conditions; 2) the local efficiency, global efficiency and wiring-cost monotonously increased, but modularity and robustness monotonously decreased in the brain networks from RS to VSW, and then to VSD; and 3) the increased nodal efficiency was highly associated with visual and working memory-related regions when performing visual tasks.

### Changes in global topologies

In the present study, we characterized the small-world properties and modularity of the brain functional networks in three different cognitive conditions, RS, VSW, and VSD. Small-worldness facilitates the maintenance of highly effective and specialized modular information processing and rapid global information transfer [8, 45], and modularity facilitates the rapid reorganization of a network through alterations in the functionality of one module without losing functionality in other modules [11]. The observations in the present study suggest that the human brain possesses adequate flexibility to support efficient information transfer in both modulated and distributed processing, regardless of various conditions.

Although the small-world properties were qualitatively obtained in these functional networks, we observed the alteration of network topology properties, i.e., both the local and global efficiency and the wiring-cost were increased, but the modularity was decreased, in brain networks from RS to VSW, and then to VSD. In the present study, the complexity of visual tasks was rationally assumed to increase from RS to VSW, and then to VSD. Thus, the changes in network properties across the three conditions might reflect the reconfiguration of brain responses to the complexity of the visual task. More importantly, we observed that the changes in the network metrics were inherently consistent. For example, both the cluster coefficient and local efficiency describe how information is efficiently transferred locally [7], and the results of the present study suggested that both of these parameters were increased with the increasing task complexity (Fig 2). Similarly, both the characteristic path length and global efficiency describe how information is efficiently transferred globally [7]. Accordingly, the results shown in Fig 2 confirmed that the global efficiency and average shortest path length exhibited opposite alterations.

These results indicated that the greater of the task complexity, the larger the values of local and global efficiency in brain networks (Fig 2), suggesting that the values of local and global efficiency reflect the complexity of visual tasks. Correspondingly, previous studies [25] have shown that the brain exhibits higher global efficiency with increasing task difficulty in subjects performing working memory tasks. In addition, in the present study, the modularity of brain functional network was reduced with increasing of task complexity (Fig 3), accompanying increased network efficiency. This observation might suggest that the segregation of the functional brain is reduced in subjects performing a complex task. Thus, these results suggested that the intra-module connections might be decreased and the inter-module connections might be increased during complex cognitive states [25]. This result is consistent with the notion that the dynamic connectivity between distinct brain systems occurs only upon task demands [1].

The brain network has been characterized as an economical trade-off between minimizing wiring-cost and maximizing topological efficiency [1]. According to this notion, brain wiring-costs are low but not minimal [46]; if the costs were minimal, then the brain would be topologically arranged as a lattice [47], and the brain efficiency would be too low to sustain any cognitive state. Thus, if the efficiency of the brain network increases, then the wiring-costs must also be increased. Our findings that wiring-costs increase (i.e., increased average degree and physical distance) with increasing visual task complexity (Fig 3) and the increased values of local and global efficiency are consistent with this notion. Altogether, we suggest that the brain functional network reorganizes through the adoption of a more efficient, but more costly, network configuration when there is greater demand for cognitive processing [1].

In particular, in the RS condition, subjects were required to close their eyes and just to keep relax, which reflected the baseline state of brain function. In the VSW condition, subjects were required to watch the stimuli only, which induced the brain to perform the visual function. Whereas in the VSD condition, subjects were required not only to watch the stimuli but also to make judgment to one-back working memory, which may induce both visual and memorial functions. Thus, the task complexity related to the involved brain functions (e.g., the visual processing and the working memory) is distinct across these three conditions. Consistent with the increase of task complexity, we found that the functional network topological properties were altered relative to the addition of the task demands in the three conditions. Moreover, we detected brain regions with significantly increased nodal efficiency following with the task demands, and these regions are highly located in the visual and memory related modules. This observation provided experimental evidence for the inherent relationship between the changed functional network properties (global and nodal parameters) and the task demands. Thereby, the change of brain functional network topological properties in the present study might be one of the potential underpinnings for the change of brain functions.

### Changes in the regional topologies

In addition to the global topologies, the current study also revealed significant between-condition differences in nodal efficiency. In several regions, the nodal efficiency increased with increasing visual task complexity (Fig 4). The increased nodal efficiency might reflect increased global topologies. The mental processes underlying VSW and VSD might be different, as VSW is a simple visual task and VSD involves both working memory and visual tasks. Based on these visual tasks, we showed that the changes in the nodal topologies in the brain functional network depend on the specific task type [48]. Many neuroimaging studies have shown that visual tasks primarily involve the occipital regions [49, 50], and working memory tasks primarily involve the default mode network, fronto-parietal control network, and occipital- and cerebellum-related regions [51, 52]. Consistent with these findings, we also observed a similar distribution of brain regions responding to the VSW and VSD tasks compared with the resting-state (Fig 4). Thus, we proposed that the alterations in the global topologies in brain networks are closely modulated through the visual task, associated with the task demands, whereas alterations in the regional topologies of specific brain areas are not simply affected by task complexity, but also restricted with task types [53].

Although our results suggested the increased nodal efficiency was highly associated with visual and working memory related regions, we cannot exclude the possible influence of other nodes on the change of nodal efficiency for a specific node. Notably, this study tried to manipulate the mental process, including the visual and working memory with different tasks, to explore the functional network topological reconfiguration following the increased task demand. Herein, the focus of the present study lies on the inter-regional communication within the brain functional network, although we determined brain regions with significantly changed nodal parameters. We noted that the nodal properties determined from graph analysis have already included the interactions among brain nodes [7]. Thereby, several potential possibilities should be considered to give an interpretation of this finding. A possible explanation is that the increased task complexity might enhance the cooperation of all nodes within brain network, which further triggered the increased nodal efficiency in the visual and working memory regions we observed.

### Validation analysis

The validation analysis showed that the results of the network analysis based on the AAL90 template exhibited low reproducibility (Table 2), potentially indicating the incompatibility of the anatomical template for functional data [54]. Previous studies [28, 54] have suggested that the use of coarse anatomic parcellation schemes for functional data might be detrimental to the quality of the constructed brain network, at least for studies focusing on global and regional network properties. A recent study showed that the functional template had higher test-retest reliabilities for network properties relative to the AAL atlas [6]. Thus, it was reasonable to use a functional template in the present study. Furthermore, we repeated the network analysis using a high-resolution functional template (i.e., the Fun268) and weighted network, which showed high reproducibility of the network result (Table 2), suggesting that these findings are not attributed to random noise, but indeed represent the inherent effects of task demands on the topological organization of brain functional networks.

### Limitations

There were several limitations in the present study, which should be addressed in future work. First, the levels of task complexity might bias the results. Notably, the three tasks in the present study were easy, with a low-range of visual task complexity for healthy volunteers. Given that the efficiency of the brain network cannot be infinitely enhanced [1] when task complexity is further increased at a high range, the relationship between the demands of the visual task and the brain network efficiency might not conform to a linear relationship. Thus, further studies should use higher levels of task difficulty to provide a more precise description. Second, the selected band frequency might influence the results of the network analysis. In the present study, we used 0.01 Hz high-pass filtering to ensure a consistent frequency among the three tasks. However, previous studies have demonstrated that brain functional organization is specific to the band frequency [55, 56], and changes in the network topology are also frequency-dependent [39]. Therefore, the effect of different band frequencies on the findings presented herein might be an interesting topic for future study. Third, the alternative methodologies might affect the final results of the complex network analysis. Previous studies have indicated that the network analysis is affected by strategies of removing the head motion artifact in fMRI data [57–59], node definition approaches [31, 60], thresholding procedures [61, 62], and null models [63, 64]. However, there remains no consistent agreement on standard data processing procedures. Thus, future studies should address whether these factors affect the current findings. Forth, although the findings of the present study provided new insights into the neural substrate of the brain function reconfiguration relative to task complexity, we didn’t evaluate the complexity of brain network. However, the methods to evaluate the complexity of a complex network have not been well established so far. Thus, the network complexity metric should be taken to directly explore the network topological organization in future works.

## Conclusion

In conclusion, the efficient topological organization was highly preserved in brain network underlying various cognitive conditions; however, information communication was also modulated to the demands of visual task. Specially, the brain functional network reorganizes through the adoption of a more efficient, but more costly, less modular, and less economical, configuration to sustain greater visual task complexity. The reconfiguration of brain network regional topologies is also affected through task complexity. The present study may provide further insight into the neural basis of the adaptively dynamic reorganization of the brain functional network induced through distinct cognitive conditions.

## Supporting Information

S1 Appendix Supplementary Materials. (DOC)
