# Supplementary material for: Reconfiguration of the Brain Functional Network Associated with Visual Task Demands
Source: PLoS One. 2015 Jul 6;10(7):e0132518. doi: 10.1371/journal.pone.0132518 (PMC4493060; doi:10.1371/journal.pone.0132518)
Supplement: S1 Appendix — (DOC) [file pone.0132518.s001.doc]

**Supplementary Materials**

***Network Analysis***

According to a recent review , the metrics of the brain functional network include:

**Average degree, *K***, is the average of the degrees of all the nodes in a graph. This value can be used to describe the connection density or wiring-cost of a network.

$K=\frac{1}{N(N-1)/2}\sum_{i\neq j\in A} A_{ij}$, (1)

where *N* denotes the number of nodes, $\sum_{i\neq j\in A} A_{ij}$ corresponds to the total number of edges in the binary matrix *A*.

**Clustering coefficient,** $C_{p}$, measures the fraction of node neighbors that are also connected to one another. This value provides information about the connectedness of node neighbors.

$C_{p}=\frac{1}{N}\sum_{i\in G} \frac{E_{i}}{k_{i}(k_{i}-1)/2}$, (2)

where $k_{i}=\sum_{j\notin i\in G} e_{ij}$ (*e*_ij_ is the *ij*th element of the binary matrix) is the degree of node *i*, *E*_i_ is the number of edges in *G*_i_ (the subgraph of node *i*). $C_{p}$ is closely related to local efficiency, $E_{loc}$, which describes how efficiently information is transferred locally within a sub-network and reflects the fault tolerance of the network.

$E_{loc}=\frac{1}{N}\sum_{i\in G} E_{glob}(i)$, (3)

where $E_{glob}(i)$ is the global efficiency of *G*_i_ (the subgraph of node *i*), whose definition is shown in formula (5) in the following section.

**Characteristic** **path length,** $L_{p}$, is the average shortest path length (the smallest number of connections that must be traversed from one node to the destination node) between all pairs of nodes in the network.

$L_{p}=\frac{1}{\frac{1}{N(N-1)}(\sum_{j\neq i\in G} \frac{1}{L_{ij}})}$ , (4)

where $L_{ij}$ is the shortest path length between nodes *i* and *j*. $L_{p}$ is closely related to global efficiency, $E_{glob}$, which is the inverse of average shortest path length. Global efficiency describes how well information is transferred globally.

$E_{glob}=\frac{1}{N(N-1)}\sum_{j\neq i\in G} \frac{1}{L_{ij}}$ (5)

**Small-worldness,** $\sigma=\gamma/\lambda$, is an index characterizing the small-world properties of a network. The normalized clustering coefficient is $\gamma=C_{p}^{real}/C_{p}^{rand}$, and the normalized characteristic path length is $\lambda=L_{p}^{real}/L_{p}^{rand}$, where $C_{p}^{real}$ and $L_{p}^{real}$ are the clustering coefficient and characteristic path length of real network, respectively. $C_{p}^{rand}$ and $L_{p}^{rand}$ are obtained from a set of 100 comparable random networks with the same number of nodes and edges as the real network. If $\sigma$> 1, then the network has small-world properties, and the larger $\sigma$ value corresponds to stronger small-world properties.

**Average physical distance,** $D_{p}$, is the average physical distance over all edges (i.e., functional connections) in the graph . The physical distance of an edge in the resulting graph is simply estimated as the Euclidean distance between the centroids of the two graphically connected brain regions in a standard MNI stereotactic space. Similar to the average degree, $D_{p}$ can also be used to describe the wiring-cost of a network but from the view of physical connected distance.

**Modularity, *Q***, is the possibility of a network being subdivided into a set of non-overlapping modules containing dense intra-module connections and relatively sparse inter-module connections of nodes , given by

$Q=\sum_{i=1}^{N_{M}} \left[\frac{e_{ii}}{M}-\left(\frac{a_{i}}{M}\right)^{2}\right]$, (6)

where *N*_M_ is the number of modules, *e*_ii_ is a measure of intra-modular edges in module *i*, *a*_i_ is the total degree of module *i*, and *M* is equal to the degree of the entire network. This function was maximized using a computationally expedient greedy algorithm .

**Nodal efficiency, *E*_nod_**, is the inverse of the mean harmonic shortest path length between one node and all other nodes in the network, defined as

$E_{nod}\left(i\right)=\frac{1}{N_{G_{i}}(N_{G_{i}}-1)}\sum_{j,k\in G_{i}} \frac{1}{L_{j,k}}$, (7)

where $N_{G_{i}}$ is the number of nodes in the *G*_i_ (the subgraph of node *i*), $L_{j,k}$ is the shortest path length between nodes *j* and *k*.

***Validation Analysis***

Further network analysis was applied to validate the reproducibility of the results: (1) Brain template parcellation effects. Graph-theory based brain network analyses are sensitive to the choice of parcellation schemes . Specially, the results of anatomical templates were shown to be different from those of functional templates . Thus, we applied a commonly employed anatomical atlas, the automated anatomical atlas (AAL90, the number of nodes N = 90) , to evaluate the effects of different nodal templates. Considering that the number of regions in a template plays an important role on the network properties, we applied the same functional template, but with higher resolution (Fun268, the number of nodes N = 268) to evaluate the effect of parcellation resolution. The information for these regions is listed in Tables B and C. (2) Effects of network type. Although a binary network is simple and easy to interpret, the characterization of brain network organization is less accurate than a weighted network . Thus, we also validated the binary network properties compared with those of the weighted network in the present study. Briefly, after changing the correlation matrix into a binary matrix using a threshold, we obtained a weighted network using the dot product of the original correlation matrix and the corresponding binary matrix. For these calculations, the validation analyses focused on the global parameters and network robustness.

Figure A. The flow chart of experimental paradigm for VSW and VSD. VSW: visual stimulus watching task, VSD: visual stimulus decision task.


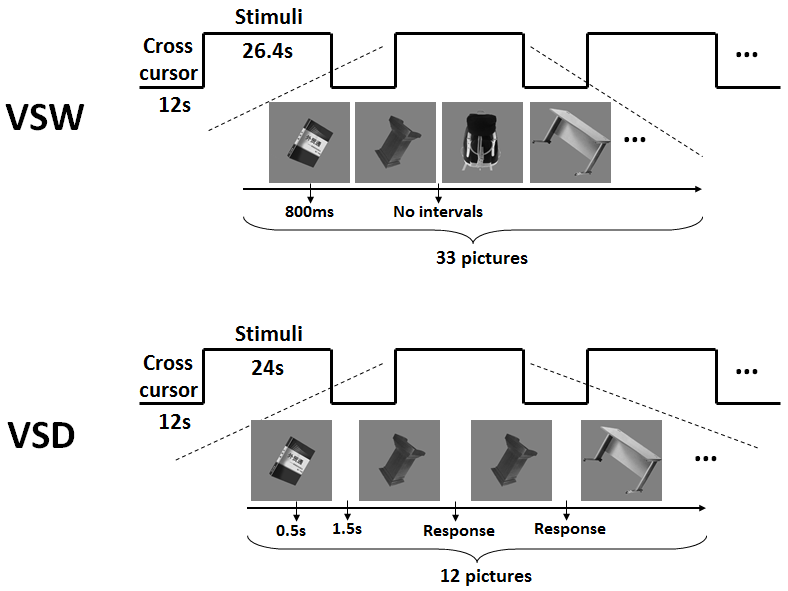


**Table A.** The names of the 160 functional nodes and their corresponding MNI coordinates in the Fun160 template according to Dosenbach et al. .

| **Regions** | **Subnetwork** | **MNI-coordinates** | | | **Regions** | **Subnetwork** | **MNI-coordinates** | | |
| --- | --- | --- | --- | --- | --- | --- | --- | --- | --- |
|  |  | **x(mm)** | **y(mm)** | **z(mm)** |  |  | **x(mm)** | **y(mm)** | **z(mm)** |
| vmPFC | default | 6 | 64 | 3 | parietal | cingulo-opercular | 58 | -41 | 20 |
| mPFC | default | 0 | 51 | 32 | temporal | cingulo-opercular | 43 | -43 | 8 |
| aPFC | default | -25 | 51 | 27 | parietal | cingulo-opercular | -55 | -44 | 30 |
| vmPFC | default | 9 | 51 | 16 | sup temporal | cingulo-opercular | 42 | -46 | 21 |
| vmPFC | default | -6 | 50 | -1 | angular gyrus | cingulo-opercular | -41 | -47 | 29 |
| vmPFC | default | -11 | 45 | 17 | temporal | cingulo-opercular | -59 | -47 | 11 |
| vmPFC | default | 8 | 42 | -5 | TPJ | cingulo-opercular | -52 | -63 | 15 |
| ACC | default | 9 | 39 | 20 | frontal | sensorimotor | 58 | 11 | 14 |
| vlPFC | default | 46 | 39 | -15 | dFC | sensorimotor | 60 | 8 | 34 |
| sup frontal | default | 23 | 33 | 47 | vFC | sensorimotor | -55 | 7 | 23 |
| sup frontal | default | -16 | 29 | 54 | pre-SMA | sensorimotor | 10 | 5 | 51 |
| inf temporal | default | 52 | -15 | -13 | vFC | sensorimotor | 43 | 1 | 12 |
| inf temporal | default | -59 | -25 | -15 | SMA | sensorimotor | 0 | -1 | 52 |
| post cingulate | default | 1 | -26 | 31 | frontal | sensorimotor | 53 | -3 | 32 |
| fusiform | default | 28 | -37 | -15 | precentral gyrus | sensorimotor | 58 | -3 | 17 |
| precuneus | default | -3 | -38 | 45 | mid insula | sensorimotor | -42 | -3 | 11 |
| post cingulate | default | -8 | -41 | 3 | precentral gyrus | sensorimotor | -44 | -6 | 49 |
| inf temporal | default | -61 | -41 | -2 | parietal | sensorimotor | -26 | -8 | 54 |
| occipital | default | -28 | -42 | -11 | precentral gyrus | sensorimotor | 46 | -8 | 24 |
| post cingulate | default | -5 | -43 | 25 | precentral gyrus | sensorimotor | -54 | -9 | 23 |
| precuneus | default | 9 | -43 | 25 | precentral gyrus | sensorimotor | 44 | -11 | 38 |
| precuneus | default | 5 | -50 | 33 | parietal | sensorimotor | -47 | -12 | 36 |
| post cingulate | default | -5 | -52 | 17 | mid insula | sensorimotor | 33 | -12 | 16 |
| post cingulate | default | 10 | -55 | 17 | mid insula | sensorimotor | -36 | -12 | 15 |
| precuneus | default | -6 | -56 | 29 | temporal | sensorimotor | 59 | -13 | 8 |
| post cingulate | default | -11 | -58 | 17 | parietal | sensorimotor | -38 | -15 | 59 |
| angular gyrus | default | 51 | -59 | 34 | parietal | sensorimotor | -47 | -18 | 50 |
| angular gyrus | default | -48 | -63 | 35 | parietal | sensorimotor | 46 | -20 | 45 |
| precuneus | default | 11 | -68 | 42 | parietal | sensorimotor | -55 | -22 | 38 |
| IPS | default | -36 | -69 | 40 | precentral gyrus | sensorimotor | -54 | -22 | 22 |
| occipital | default | -9 | -72 | 41 | temporal | sensorimotor | -54 | -22 | 9 |
| occipital | default | 45 | -72 | 29 | parietal | sensorimotor | 41 | -23 | 55 |
| occipital | default | -2 | -75 | 32 | post insula | sensorimotor | 42 | -24 | 17 |
| occipital | default | -42 | -76 | 26 | parietal | sensorimotor | 18 | -27 | 62 |
| aPFC | fronto-parietal | 29 | 57 | 18 | parietal | sensorimotor | -38 | -27 | 60 |
| aPFC | fronto-parietal | -29 | 57 | 10 | parietal | sensorimotor | -24 | -30 | 64 |
| vent aPFC | fronto-parietal | 42 | 48 | -3 | post parietal | sensorimotor | -41 | -31 | 48 |
| vent aPFC | fronto-parietal | -43 | 47 | 2 | temporal | sensorimotor | -41 | -37 | 16 |
| vlPFC | fronto-parietal | 39 | 42 | 16 | temporal | sensorimotor | -53 | -37 | 13 |
| dlPFC | fronto-parietal | 40 | 36 | 29 | sup parietal | sensorimotor | 34 | -39 | 65 |
| ACC | fronto-parietal | -1 | 28 | 40 | occipital | occipital | -18 | -50 | 1 |
| dlPFC | fronto-parietal | 46 | 28 | 31 | occipital | occipital | -34 | -60 | -5 |
| vPFC | fronto-parietal | -52 | 28 | 17 | occipital | occipital | 36 | -60 | -8 |
| dlPFC | fronto-parietal | -44 | 27 | 33 | temporal | occipital | 46 | -62 | 5 |
| dFC | fronto-parietal | 40 | 17 | 40 | occipital | occipital | -44 | -63 | -7 |
| dFC | fronto-parietal | 44 | 8 | 34 | occipital | occipital | 19 | -66 | -1 |
| dFC | fronto-parietal | -42 | 7 | 36 | occipital | occipital | 17 | -68 | 20 |
| IPL | fronto-parietal | -41 | -40 | 42 | occipital | occipital | 39 | -71 | 13 |
| IPL | fronto-parietal | 54 | -44 | 43 | occipital | occipital | 29 | -73 | 29 |
| post parietal | fronto-parietal | -35 | -46 | 48 | occipital | occipital | -29 | -75 | 28 |
| IPL | fronto-parietal | -48 | -47 | 49 | occipital | occipital | -16 | -76 | 33 |
| IPL | fronto-parietal | -53 | -50 | 39 | occipital | occipital | 9 | -76 | 14 |
| IPL | fronto-parietal | 44 | -52 | 47 | occipital | occipital | 15 | -77 | 32 |
| IPS | fronto-parietal | -32 | -58 | 46 | occipital | occipital | 20 | -78 | -2 |
| IPS | fronto-parietal | 32 | -59 | 41 | post occipital | occipital | -5 | -80 | 9 |
| aPFC | cingulo-opercular | 27 | 49 | 26 | post occipital | occipital | 29 | -81 | 14 |
| vPFC | cingulo-opercular | 34 | 32 | 7 | post occipital | occipital | 33 | -81 | -2 |
| ACC | cingulo-opercular | -2 | 30 | 27 | post occipital | occipital | -37 | -83 | -2 |
| vFC | cingulo-opercular | 51 | 23 | 8 | post occipital | occipital | -29 | -88 | 8 |
| ant insula | cingulo-opercular | 38 | 21 | -1 | post occipital | occipital | 13 | -91 | 2 |
| dACC | cingulo-opercular | 9 | 20 | 34 | post occipital | occipital | 27 | -91 | 2 |
| ant insula | cingulo-opercular | -36 | 18 | 2 | post occipital | occipital | -4 | -94 | 12 |
| basal ganglia | cingulo-opercular | -6 | 17 | 34 | lat cerebellum | cerebellum | -28 | -44 | -25 |
| mFC | cingulo-opercular | 0 | 15 | 45 | lat cerebellum | cerebellum | -24 | -54 | -21 |
| vFC | cingulo-opercular | -46 | 10 | 14 | inf cerebellum | cerebellum | -37 | -54 | -37 |
| basal ganglia | cingulo-opercular | -20 | 6 | 7 | lat cerebellum | cerebellum | -34 | -57 | -24 |
| basal ganglia | cingulo-opercular | 14 | 6 | 7 | med cerebellum | cerebellum | -6 | -60 | -15 |
| vFC | cingulo-opercular | -48 | 6 | 1 | inf cerebellum | cerebellum | -25 | -60 | -34 |
| mid insula | cingulo-opercular | 37 | -2 | -3 | inf cerebellum | cerebellum | 32 | -61 | -31 |
| thalamus | cingulo-opercular | -12 | -3 | 13 | med cerebellum | cerebellum | -16 | -64 | -21 |
| thalamus | cingulo-opercular | -12 | -12 | 6 | lat cerebellum | cerebellum | 21 | -64 | -22 |
| thalamus | cingulo-opercular | 11 | -12 | 6 | med cerebellum | cerebellum | 1 | -66 | -24 |
| mid insula | cingulo-opercular | 32 | -12 | 2 | inf cerebellum | cerebellum | -34 | -67 | -29 |
| mid insula | cingulo-opercular | -30 | -14 | 1 | med cerebellum | cerebellum | -11 | -72 | -14 |
| basal ganglia | cingulo-opercular | 11 | -24 | 2 | inf cerebellum | cerebellum | 33 | -73 | -30 |
| post insula | cingulo-opercular | -30 | -28 | 9 | med cerebellum | cerebellum | 5 | -75 | -11 |
| temporal | cingulo-opercular | 51 | -30 | 5 | med cerebellum | cerebellum | 14 | -75 | -21 |
| post cingulate | cingulo-opercular | -4 | -31 | -4 | inf cerebellum | cerebellum | -21 | -79 | -33 |
| fusiform | cingulo-opercular | 54 | -31 | -18 | inf cerebellum | cerebellum | -6 | -79 | -33 |
| precuneus | cingulo-opercular | 8 | -40 | 50 | inf cerebellum | cerebellum | 18 | -81 | -33 |

**Table B.** The names of the 90 anatomical nodes and their respective mask labels in the AAL90 atlas according to Tzourio-Mazoyer et al. .

| **Anatomical region** | **AAL mask label** | **Anatomical region** | **AAL mask label** |
| --- | --- | --- | --- |
| Left anterior cingulate cortex | L.ACC | Right anterior cingulate cortex | R.ACC |
| Left amygdala | L.AMY | Right amygdala | R.AMY |
| Left angular gyrus | L.ANG | Right angular gyrus | R.ANG |
| Left calcarine sulcus | L.CAL | Right calcarine sulcus | R.CAL |
| Left caudate | L.CAU | Right caudate | R.CAU |
| Left cuneus | L.CUN | Right cuneus | R.CUN |
| Left fusiform gyrus | L.FUS | Right fusiform gyrus | R.FUS |
| Left heschl’sgyrus | L.HES | Right heschl’sgyrus | R.HES |
| Left hippocampus | L.HIP | Right hippocampus | R.HIP |
| Left inferior occipital gyrus | L.IOG | Right inferior occipital gyrus | R.IOG |
| Left inferior parietal lobe | L.IPL | Right inferior parietal lobe | R.IPL |
| Left inferior temporal gyrus | L.ITG | Right inferior temporal gyrus | R.ITG |
| Left insula | L.INS | Right insula | R.INS |
| Left lingual gyrus | L.LIN | Right lingual gyrus | R.LIN |
| Left middle cingulate cortex | L.MCC | Right middle cingulate cortex | R.MCC |
| Left superior frontal gyrus (medial) | L.MedSFG | Right superior frontal gyrus (medial) | R.MedSFG |
| Left mid frontal gyrus | L.MFG | Right mid frontal gyrus | R.MFG |
| Left middle occipital gyrus | L.MOG | Right middle occipital gyrus | R.MOG |
| Left middle temporal pole | L.MidTP | Right middle temporal pole | R.MidTP |
| Left middle temporal gyrus | L.MTG | Right middle temporal gyrus | R.MTG |
| Left olfactory cortex | L.OLF | Right olfactory cortex | R.OLF |
| Left inferior frontal gyrus (operculum) | L.OperIFG | Right inferior frontal gyrus (operculum) | R.OperIFG |
| Left rolandic operculum | L.ROL | Right rolandic operculum | R.ROL |
| Left inferior frontal gyrus (orbital) | L.OrbIFG | Right inferior frontal gyrus (orbital) | R.OrbIFG |
| Left medial orbitofrontal cortex | L.OMF | Right medial orbitofrontal cortex | R.OMF |
| Left middle frontal gyrus (orbital) | L.OrbMFG | Right middle frontal gyrus (orbital) | R.OrbMFG |
| Left superior frontal gyrus (orbital) | L.OrbSFG | Right superior frontal gyrus (orbital) | R.OrbSFG |
| Left pallidum | L.PAL | Right pallidum | R.PAL |
| Left paracentral lobule | L.PCL | Right paracentral lobule | R.PCL |
| Left parahippocampalgyrus | L.PHP | Right parahippocampalgyrus | R.PHP |
| Left posterior cingulate cortex | L.PCC | Right posterior cingulate cortex | R.PCC |
| Left postcentralgyrus | L.PostCG | Right postcentralgyrus | R.PostCG |
| Left precentralgyrus | L.PreCG | Right precentralgyrus | R.PreCG |
| Left precuneus | L.PreCUN | Right precuneus | R.PreCUN |
| Left putamen | L.PUT | Right putamen | R.PUT |
| Left rectus gyrus | L.REC | Right rectus gyrus | R.REC |
| Left supplementary motor area | L.SMA | Right supplementary motor area | R.SMA |
| Left superior frontal gyrus | L.SFG | Right superior frontal gyrus | R.SFG |
| Left superior occipital gyrus | L.SOG | Right superior occipital gyrus | R.SOG |
| Left superior parietal lobe | L.SPL | Right superior parietal lobe | R.SPL |
| Left superior temporal pole | L.SupTP | Right superior temporal pole | R.SupTP |
| Left superior temporal gyrus | L.STG | Right superior temporal gyrus | R.STG |
| Left supramarginalgyrus | L.SMG | Right supramarginalgyrus | R.SMG |
| Left thalamus | L.THA | Right thalamus | R.THA |
| Left inferior frontal gyrus (triangular) | L.TriIFG | Right inferior frontal gyrus (triangular) | R.TriIFG |

**Table C.** The names of the 268 functional nodes and their corresponding MNI coordinates in the Fun268 template according to Cao et al. .

| **Regions** | **MNI-coordinates** | | | **Regions** | **MNI-coordinates** | | |
| --- | --- | --- | --- | --- | --- | --- | --- |
|  | **x(mm)** | **y(mm)** | **z(mm)** |  | **x(mm)** | **y(mm)** | **z(mm)** |
| Occipital_Inf_L | -25 | -98 | -12 | Precuneus_R | 11 | -66 | 42 |
| Occipital_Inf_R | 27 | -97 | -13 | Precuneus_R | 4 | -48 | 51 |
| Frontal_Sup_Orb_R | 24 | 32 | -18 | Frontal_Inf_Orb_L | -46 | 31 | -13 |
| Temporal_Inf_L | -56 | -45 | -24 | Supp_Motor_Area_L | -10 | 11 | 67 |
| Rectus_R | 8 | 41 | -24 | Frontal_Inf_Orb_R | 49 | 35 | -12 |
| ParaHippocampal_L | -21 | -22 | -20 | Lingual_R | 8 | -91 | -7 |
| ParaHippocampal_R | 17 | -28 | -17 | Lingual_R | 17 | -91 | -14 |
| Fusiform_L | -37 | -29 | -26 | Lingual_L | -12 | -95 | -13 |
| Temporal_Mid_R | 65 | -24 | -19 | Lingual_R | 18 | -47 | -10 |
| Temporal_Inf_R | 52 | -34 | -27 | Occipital_Mid_R | 40 | -72 | 14 |
| Temporal_Inf_R | 55 | -31 | -17 | Calcarine_R | 8 | -72 | 11 |
| Frontal_Inf_Orb_R | 34 | 38 | -12 | Calcarine_L | -8 | -81 | 7 |
| Precuneus_L | -7 | -52 | 61 | Occipital_Mid_L | -28 | -79 | 19 |
| Cingulum_Mid_L | -14 | -18 | 40 | Lingual_R | 20 | -66 | 2 |
| Supp_Motor_Area_L | 0 | -15 | 47 | Occipital_Mid_L | -24 | -91 | 19 |
| Supp_Motor_Area_R | 10 | -2 | 45 | Fusiform_R | 27 | -59 | -9 |
| Paracentral_Lobule_L | -7 | -21 | 65 | Lingual_L | -15 | -72 | -8 |
| Paracentral_Lobule_L | -7 | -33 | 72 | Calcarine_L | -18 | -68 | 5 |
| Postcentral_R | 13 | -33 | 75 | Occipital_Inf_R | 43 | -78 | -12 |
| Parietal_Inf_L | -54 | -23 | 43 | Occipital_Inf_L | -47 | -76 | -10 |
| Precentral_R | 29 | -17 | 71 | Occipital_Sup_L | -14 | -91 | 31 |
| Precuneus_R | 10 | -46 | 73 | Cuneus_R | 15 | -87 | 37 |
| Postcentral_L | -23 | -30 | 72 | Occipital_Mid_R | 29 | -77 | 25 |
| Precentral_L | -40 | -19 | 54 | Lingual_R | 20 | -86 | -2 |
| Postcentral_R | 29 | -39 | 59 | Cuneus_R | 15 | -77 | 31 |
| Postcentral_R | 50 | -20 | 42 | Lingual_L | -16 | -52 | -1 |
| Precentral_L | -38 | -27 | 69 | Temporal_Inf_R | 42 | -66 | -8 |
| Postcentral_R | 20 | -29 | 60 | Occipital_Sup_R | 24 | -87 | 24 |
| Precentral_R | 44 | -8 | 57 | Cuneus_R | 6 | -72 | 24 |
| Postcentral_L | -29 | -43 | 61 | Occipital_Mid_L | -42 | -74 | 0 |
| Supp_Motor_Area_R | 10 | -17 | 74 | Fusiform_R | 26 | -79 | -16 |
| Postcentral_R | 22 | -42 | 69 | Cuneus_L | -16 | -77 | 34 |
| Postcentral_L | -45 | -32 | 47 | Cuneus_L | -3 | -81 | 21 |
| Postcentral_L | -21 | -31 | 61 | Occipital_Mid_L | -40 | -88 | -6 |
| Paracentral_Lobule_L | -13 | -17 | 75 | Occipital_Mid_R | 37 | -84 | 13 |
| Precentral_R | 42 | -20 | 55 | Calcarine_R | 6 | -81 | 6 |
| Precentral_L | -38 | -15 | 69 | Occipital_Mid_L | -26 | -90 | 3 |
| Parietal_Sup_L | -16 | -46 | 73 | Occipital_Inf_L | -33 | -79 | -13 |
| Paracentral_Lobule_R | 2 | -28 | 60 | Occipital_Mid_R | 37 | -81 | 1 |
| Supp_Motor_Area_R | 3 | -17 | 58 | Precentral_L | -44 | 2 | 46 |
| Precentral_R | 38 | -17 | 45 | Frontal_Inf_Tri_R | 48 | 25 | 27 |
| Postcentral_L | -49 | -11 | 35 | Frontal_Inf_Tri_L | -47 | 11 | 23 |
| Insula_R | 36 | -9 | 14 | Parietal_Inf_L | -53 | -49 | 43 |
| Postcentral_R | 51 | -6 | 32 | Frontal_Sup_L | -23 | 11 | 64 |
| Postcentral_L | -53 | -10 | 24 | Temporal_Inf_R | 58 | -53 | -14 |
| Postcentral_R | 66 | -8 | 25 | Frontal_Sup_Orb_R | 24 | 45 | -15 |
| Supp_Motor_Area_L | -3 | 2 | 53 | Frontal_Mid_Orb_R | 34 | 54 | -13 |
| SupraMarginal_R | 54 | -28 | 34 | Frontal_Mid_Orb_L | -21 | 41 | -20 |
| Frontal_Sup_R | 19 | -8 | 64 | Cerebelum_L | -18 | -76 | -24 |
| Frontal_Sup_L | -16 | -5 | 71 | Cerebelum_R | 17 | -80 | -34 |
| Cingulum_Mid_L | -10 | -2 | 42 | Cerebelum_R | 35 | -67 | -34 |
| Claustrum_R | 37 | 1 | -4 | Precentral_R | 47 | 10 | 33 |
| Supp_Motor_Area_R | 13 | -1 | 70 | Precentral_L | -41 | 6 | 33 |
| Supp_Motor_Area_R | 7 | 8 | 51 | Frontal_Mid_L | -42 | 38 | 21 |
| Rolandic_Oper_L | -45 | 0 | 9 | Frontal_Mid_R | 38 | 43 | 15 |
| Rolandic_Oper_R | 49 | 8 | -1 | Parietal_Inf_R | 49 | -42 | 45 |
| Claustrum_L | -34 | 3 | 4 | Parietal_Inf_L | -28 | -58 | 48 |
| Temporal_Pole_Sup_L | -51 | 8 | -2 | Parietal_Inf_R | 44 | -53 | 47 |
| Cingulum_Mid_L | -5 | 18 | 34 | Frontal_Mid_R | 32 | 14 | 56 |
| Claustrum_R | 36 | 10 | 1 | Angular_R | 37 | -65 | 40 |
| Insula_R | 32 | -26 | 13 | Parietal_Inf_L | -42 | -55 | 45 |
| Temporal_Sup_R | 65 | -33 | 20 | Frontal_Mid_R | 40 | 18 | 40 |
| Temporal_Sup_R | 58 | -16 | 7 | Frontal_Mid_L | -34 | 55 | 4 |
| Rolandic_Oper_L | -38 | -33 | 17 | Frontal_Mid_Orb_L | -42 | 45 | -2 |
| SupraMarginal_L | -60 | -25 | 14 | Parietal_Inf_R | 33 | -53 | 44 |
| Temporal_Sup_L | -49 | -26 | 5 | Frontal_Mid_Orb_R | 43 | 49 | -2 |
| Rolandic_Oper_R | 43 | -23 | 20 | Frontal_Inf_Tri_L | -42 | 25 | 30 |
| SupraMarginal_L | -50 | -34 | 26 | Frontal_Sup_Medial_L | -3 | 26 | 44 |
| SupraMarginal_L | -53 | -22 | 23 | Cingulum_Mid_R | 11 | -39 | 50 |
| Rolandic_Oper_L | -55 | -9 | 12 | SupraMarginal_R | 55 | -45 | 37 |
| Rolandic_Oper_R | 56 | -5 | 13 | Precentral_R | 42 | 0 | 47 |
| Postcentral_R | 59 | -17 | 29 | Frontal_Mid_R | 31 | 33 | 26 |
| Insula_L | -30 | -27 | 12 | Frontal_Inf_Tri_R | 48 | 22 | 10 |
| Occipital_Mid_L | -41 | -75 | 26 | Insula_L | -35 | 20 | 0 |
| Frontal_Med_Orb_R | 6 | 67 | -4 | Insula_R | 36 | 22 | 3 |
| Frontal_Med_Orb_R | 8 | 48 | -15 | Frontal_Inf_Orb_R | 37 | 32 | -2 |
| Precuneus_L | -13 | -40 | 1 | Insula_R | 34 | 16 | -8 |
| Frontal_Sup_Orb_L | -18 | 63 | -9 | Cingulum_Ant_L | -11 | 26 | 25 |
| Temporal_Mid_L | -46 | -61 | 21 | Supp_Motor_Area_L | -1 | 15 | 44 |
| Occipital_Mid_R | 43 | -72 | 28 | Frontal_Mid_L | -28 | 52 | 21 |
| Temporal_Pole_Mid_L | -44 | 12 | -34 | Cingulum_Ant_L | 0 | 30 | 27 |
| Temporal_Pole_Mid_R | 46 | 16 | -30 | Cingulum_Mid_R | 5 | 23 | 37 |
| Temporal_Mid_L | -68 | -23 | -16 | Cingulum_Ant_R | 10 | 22 | 27 |
| Temporal_Mid_L | -58 | -26 | -15 | Frontal_Mid_R | 31 | 56 | 14 |
| Insula_R | 27 | 16 | -17 | Frontal_Mid_R | 26 | 50 | 27 |
| Angular_L | -44 | -65 | 35 | Frontal_Mid_L | -39 | 51 | 17 |
| Angular_L | -39 | -75 | 44 | Cingulum_Mid_R | 2 | -24 | 30 |
| Precuneus_L | -7 | -55 | 27 | Thalamus_R | 6 | -24 | 0 |
| Precuneus_R | 6 | -59 | 35 | Thalamus_L | -2 | -13 | 12 |
| Precuneus_L | -11 | -56 | 16 | Thalamus_L | -10 | -18 | 7 |
| Precuneus_L | -3 | -49 | 13 | Thalamus_R | 12 | -17 | 8 |
| Cingulum_Mid_R | 8 | -48 | 31 | Thalamus_L | -5 | -28 | -4 |
| Precuneus_R | 15 | -63 | 26 | Putamen_L | -22 | 7 | -5 |
| Cingulum_Mid_L | -2 | -37 | 44 | Putamen_L | -15 | 4 | 8 |
| Precuneus_R | 11 | -54 | 17 | Putamen_R | 31 | -14 | 2 |
| Angular_R | 52 | -59 | 36 | Putamen_R | 23 | 10 | 1 |
| Frontal_Sup_R | 23 | 33 | 48 | Putamen_R | 29 | 1 | 4 |
| Frontal_Sup_Medial_L | -10 | 39 | 52 | Putamen_L | -31 | -11 | 0 |
| Frontal_Sup_L | -16 | 29 | 53 | Putamen_R | 15 | 5 | 7 |
| Frontal_Mid_L | -35 | 20 | 51 | Thalamus_R | 9 | -4 | 6 |
| Frontal_Sup_R | 22 | 39 | 39 | Temporal_Sup_R | 54 | -43 | 22 |
| Frontal_Sup_R | 13 | 55 | 38 | Temporal_Mid_L | -56 | -50 | 10 |
| Frontal_Sup_L | -10 | 55 | 39 | Temporal_Sup_L | -55 | -40 | 14 |
| Frontal_Sup_L | -20 | 45 | 39 | Temporal_Sup_R | 52 | -33 | 8 |
| Frontal_Sup_Medial_R | 6 | 54 | 16 | Temporal_Mid_R | 51 | -29 | -4 |
| Frontal_Sup_Medial_R | 6 | 64 | 22 | Temporal_Sup_R | 56 | -46 | 11 |
| Cingulum_Ant_L | -7 | 51 | -1 | Frontal_Inf_Tri_R | 53 | 33 | 1 |
| Frontal_Sup_Medial_R | 9 | 54 | 3 | Frontal_Inf_Tri_L | -49 | 25 | -1 |
| Frontal_Med_Orb_L | -3 | 44 | -9 | Cerebelum_L | -16 | -65 | -20 |
| Frontal_Med_Orb_R | 8 | 42 | -5 | Cerebelum_L | -32 | -55 | -25 |
| Cingulum_Ant_L | -11 | 45 | 8 | Cerebelum_R | 22 | -58 | -23 |
| Frontal_Sup_Medial_L | -2 | 38 | 36 | Cerebelum_R | 1 | -62 | -18 |
| Cingulum_Ant_L | -3 | 42 | 16 | Fusiform_R | 33 | -12 | -34 |
| Frontal_Sup_L | -20 | 64 | 19 | Fusiform_L | -31 | -10 | -36 |
| Frontal_Sup_Medial_L | -8 | 48 | 23 | Temporal_Inf_R | 49 | -3 | -38 |
| Temporal_Mid_R | 65 | -12 | -19 | Temporal_Inf_L | -50 | -7 | -39 |
| Temporal_Mid_L | -56 | -13 | -10 | Precuneus_R | 10 | -62 | 61 |
| Temporal_Mid_L | -58 | -30 | -4 | Temporal_Mid_L | -52 | -63 | 5 |
| Temporal_Mid_R | 65 | -31 | -9 | Temporal_Inf_L | -47 | -51 | -21 |
| Temporal_Mid_L | -68 | -41 | -5 | Temporal_Inf_R | 46 | -47 | -17 |
| Frontal_Sup_Medial_R | 13 | 30 | 59 | Postcentral_R | 47 | -30 | 49 |
| Cingulum_Ant_R | 12 | 36 | 20 | Occipital_Sup_R | 22 | -65 | 48 |
| Temporal_Mid_R | 52 | -2 | -16 | Temporal_Mid_R | 46 | -59 | 4 |
| ParaHippocampal_L | -26 | -40 | -8 | Parietal_Sup_R | 25 | -58 | 60 |
| ParaHippocampal_R | 27 | -37 | -13 | Parietal_Inf_L | -33 | -46 | 47 |
| Fusiform_L | -34 | -38 | -16 | Occipital_Mid_L | -27 | -71 | 37 |
| Cerebelum_R | 28 | -77 | -32 | Precentral_L | -32 | -1 | 54 |
| Temporal_Pole_Mid_R | 52 | 7 | -30 | Temporal_Inf_L | -42 | -60 | -9 |
| Temporal_Mid_L | -53 | 3 | -27 | Precuneus_L | -17 | -59 | 64 |
| Angular_R | 47 | -50 | 29 | Frontal_Mid_R | 29 | -5 | 54 |
| Temporal_Mid_L | -49 | -42 | 1 | Hippocampus_L | -30 | -13 | -12 |
| Insula_L | -31 | 19 | -19 | Hippocampus_R | 30 | -4 | -12 |
| Cingulum_Post_L | -2 | -35 | 31 | Amygdala_L | -24 | -1 | -16 |
| Precuneus_L | -7 | -71 | 42 | Amygdala_R | 26 | 1 | -18 |

**References**

1. Rubinov M, Sporns O. Complex network measures of brain connectivity: uses and interpretations. NeuroImage. 2010;52(3):1059-69.

2. Alexander-Bloch AF, Vertes PE, Stidd R, Lalonde F, Clasen L, Rapoport J, et al. The anatomical distance of functional connections predicts brain network topology in health and schizophrenia. Cereb Cortex. 2013;23(1):127-38.

3. Newman M, Girvan M. Finding and evaluating community structure in networks. Physical Review E. 2004;69(2).

4. Meunier D, Achard S, Morcom A, Bullmore E. Age-related changes in modular organization of human brain functional networks. NeuroImage. 2009;44(3):715-23.

5. Bohland JW, Bokil H, Allen CB, Mitra PP. The brain atlas concordance problem: quantitative comparison of anatomical parcellations. PLoS One. 2009;4(9):e7200.

6. Fornito A, Zalesky A, Bullmore ET. Network scaling effects in graph analytic studies of human resting-state FMRI data. Front Syst Neurosci. 2010;4:22. Epub 2010/07/02.

7. Smith SM, Miller KL, Salimi-Khorshidi G, Webster M, Beckmann CF, Nichols TE, et al. Network modelling methods for FMRI. NeuroImage. 2011;54(2):875-91.

8. Tzourio-Mazoyer N, Landeau B, Papathanassiou D, Crivello F, Etard O, Delcroix N, et al. Automated anatomical labeling of activations in SPM using a macroscopic anatomical parcellation of the MNI MRI single-subject brain. NeuroImage. 2002;15(1):273-89.

9. Cao H, Plichta MM, Schafer A, Haddad L, Grimm O, Schneider M, et al. Test-retest reliability of fMRI-based graph theoretical properties during working memory, emotion processing, and resting state. NeuroImage. 2014;84:888-900.

10. Barrat A, Barthelemy M, Pastor-Satorras R, Vespignani A. The architecture of complex weighted networks. Proc Natl Acad Sci U S A. 2004;101(11):3747-52.

11. Dosenbach NU, Nardos B, Cohen AL, Fair DA, Power JD, Church JA, et al. Prediction of individual brain maturity using fMRI. Science. 2010;329(5997):1358-61.
